# Genetic and Environmental Effects on the Development of White Matter Hyperintensities in a Middle Age Twin Population

**DOI:** 10.3390/medicina58101425

**Published:** 2022-10-10

**Authors:** Amirreza Alijanpourotaghsara, David Strelnikov, Marton Piroska, Laszlo Szalontai, Bianka Forgo, Zsofia Jokkel, Alíz Persely, Anita Hernyes, Lajos Rudolf Kozak, Adam Szabo, Pal Maurovich-Horvat, Adam Domonkos Tarnoki, David Laszlo Tarnoki

**Affiliations:** 1 Medical Imaging Centre, Semmelweis University, 1082 Budapest, Hungary; 2 Department of Radiology, Faculty of Medicine and Health, Örebro University, 702 81 Örebro, Sweden

**Keywords:** white matter hyperintensities, twins, MRI, heritability, volBrain

## Abstract

*Introduction:* White matter hyperintensities (WMH) indicate white matter brain lesions in magnetic resonance imaging (MRI), which can be used as a marker for brain aging and cerebrovascular and neurodegenerative disorders. Twin studies revealed substantial but not uniform WMH heritability in elderly twins. The objective of our study was to investigate the genetic and environmental components of WMH, as well as their importance in a healthy twin population, utilizing 3T MRI scanners in a middle-aged twin population. *Methods:* Brain MRI was performed on 120 healthy adult twins from the Hungarian Twin Registry on a 3T scanner (86 monozygotic, MZ and 34 dizygotic, DZ twins; median age 50 ± 26.5 years, 72.5% female and 27.5% male). The count of WMH on FLAIR images was calculated using an automated volumetry pipeline (volBrain) and human processing. The age- and sex-adjusted MZ and DZ intra-pair correlations were determined and the total variance was decomposed into genetic, shared and unique environmental components using structural equation modeling. *Results:* Age and sex-adjusted MZ intrapair correlations were higher than DZ correlations, indicating moderate genetic influence in each lesion (rMZ = 0.466, rDZ = −0.025 for total count; rMZ = 0.482, rDZ = 0.093 for deep white matter count; rMZ = 0.739, rDZ = 0.39 for infratentorial count; rMZ = 0.573, rDZ = 0.372 for cerebellar count and rMZ = 0.473, rDZ = 0.19 for periventricular count), indicating a moderate heritability (A = 40.3%, A = 45%, A = 72.7% and A = 55.5%and 47.2%, respectively). The rest of the variance was influenced by unique environmental effects (E between 27.3% and 59.7%, respectively). *Conclusions:* The number of WMH lesions is moderately influenced by genetic effects, particularly in the infratentorial region in middle-aged twins. These results suggest that the distribution of WMH in various brain regions is heterogeneous.

## 1. Introduction

Since Magnetic Resonance Imaging (MRI) became broadly available and clinicians used it for neurological imaging, the presence of the brain white matter lesions in the form of white matter hyperintensities (WMH) on fluid-attenuated inversion recovery (FLAIR) sequences or leukoaraiosis on MRI has become more common. Different studies have reported different WMH prevalence and incidence due to various imaging techniques and the target population. Most studies targeted the older population and patients with neurological disorders such as dementia or a history of stroke. The prevalence of WMH in the young population (below 45 years old) is 25.9% and mild WMH is present in most patients (85.5%). The prevalence of WMH was higher in older patients, patients with a history of CVD and patients with neurological symptoms such as headache and dizziness [1]. The prevalence of WMH in healthy adults 44–48 years old was 50.9%, with 34.1% WMH in the subcortical regions [2]. Of healthy subjects aged between 60 to 64 years old, the prevalence of WMH was 100% in the periventricular region and 96.6% in the deep white matter regions [3]. Neurodegenerative disorders such as Alzheimer’s disease frequently occur with cerebrovascular disease in elderly patients. Cerebral small vessel disease (SVD) is the most prevalent vascular cause of dementia and a major factor in mixed dementia. Alzheimer’s disease and SVD share similar risk factors, both associated with cognitive decline and dementia. MRI WMH hyperintensities are one of the important indicators of SVD, among its other neuroimaging signs [4].

The WMH are quantifiable neurological and radiological markers indicating parenchymal changes in the brain. WMHs can be categorized into focal and multifocal lesions based on their number and volume. They are divided into four major categories based on their anatomical location: subcortical, periventricular, deep white matter and infratentorial. The WMH can demonstrate a broad spectrum of causes, from vascular, inflammatory and traumatic damage to physiological aging. Although there is a long list of diagnoses of WMH, according to clinical and pathological data, ischemia is the primary cause of these lesions. According to histopathological reports, the WMH indicates myelin refraction with preserving subcortical U fibers, astrogliosis, spongiosis, axonal loss and widened perivascular spaces [5,6].

The origin of these lesions is predominantly vascular and multiple studies have discovered a strong connection between the prevalence of WMH and vascular disorders [7,8]. Previous research has suggested that axonal loss and demyelination play a role in developing WMHs, which might result from prolonged ischemia induced by cerebral small artery disease. According to certain theories, hypoperfusion may be caused by altered cerebrovascular autoregulation, blood-brain barrier failure, or inflammation [9].

After hypertension, cerebral amyloid angiopathy (CAA) is the second most prevalent cause of cerebral hemorrhage. Recurrent cerebral bleeding, ischemic strokes and cognitive deficits have all been linked to it [8]. Studies have shown that the occurrence of WMHs can also be hereditary [10]. Genetic studies indicate a moderate to high hereditary influence on the presence of vascular risk factors such as hypertension and hypercholesterolemia. Still, it is not clear whether there is a correlation between the vascular risk factors and the presence of WMH or a shared genetic factor influencing both [11].Other studies have mentioned that environmental factors such as diabetes mellitus (DM) and blood glucose level, smoking, obesity, trauma, stress and aging play an important role in the development of WMH [7,12,13,14,15,16].

Identical twins are formed when one egg cell divides in two at a very early stage of development, while non-identical twins are formed when two fertilized eggs implant in the womb simultaneously. As a result, monozygotic twins (MZ) share almost all of their genes, while dizygotic twins (DZ) typically share half of their genes. Furthermore, the MZ and DZ twins have all of their environmental influences in common but none of their differences. Twins are unique research subjects since they facilitate distinguishing between how nature (genetics) and nurture (environment) affect human health. Twin studies have provided important insights regarding the genetic basis of complex traits [17,18]. Twin studies provide a great medium to investigate the effect of genetics versus environment and their proportion in their impact on the WMHs in the brain. Due to the high number of WMHs in the aged population and their effect, most studies have been carried out on a geriatric population. Only a few studies investigated the presence of WMHs in a relatively young or middle aged population.

In this study, we aim to evaluate the genetic components of WMH, using a middle aged healthy twin population, and the effect of environmental components on them. Using a 3T MRI scanner instead of a classic 1.5 T and processing the data with an automated volumetry pipeline provide us with more precision in comparison to the majority of previous studies.

## 2. Materials and Methods

### 2.1. Study Participants

Our study investigated 120 healthy adult asymptomatic Caucasian twins (57 pairs and 2 triplets) from the Hungarian Twin Registry [19] with no history of cerebrovascular or neurodegenerative disorders. For statistical purposes, triplets were considered as three distinct twin pairs. This resulted in a total of 63 twin pairs, 43 of which were MZ pairs and 20 DZ pairs. Two twin pairs were eliminated from the research due to missing visits, while another pair was removed due to poor imaging quality. The median age of all participants was 50 ± 26.5 years and the proportion of female to male participants was 72.5% to 27.5%, respectively. The local Ethical Committee approved the study (Semmelweis University TUKEB 189−1/2014, amended on 10 October 2016 and 7 December 2018). All twins that took part signed informed consent. The principles of the Helsinki Declaration were respected. A seven-part self-reported questionnaire was used to assess the zygosity categorization [20]. A questionnaire collected information on the participant’s history and risk factors, such as height, body weight, body mass index (BMI), smoking, hypertension, hyperlipidemia and diabetes. Former smokers were also included in the smoking group.

Exclusion criteria included immunosuppressive or immunomodulatory medication in the previous month, chemotherapy in the previous year, major surgery in the last two months, transfusion of blood or blood products in the previous two months, current pregnancy, or breastfeeding. Participants with pacemakers, implantable cardioverter-defibrillators or other implanted devices, magnetic metal foreign bodies, or claustrophobia were also eliminated. Brain MRI studies were carried out between 2016 and 2021 at the Semmelweis University Medical Imaging Centre, the Magnetic Resonance Research Centre (MRKK) in Budapest, Hungary.

### 2.2. MRI Acquisition

T1W coronal, T2W sagittal, axial trace-weighted diffusion, axial apparent diffusion coefficient, axial proton density and axial T2W dark fluid (FLAIR) images of the brain were taken as part of the study. There was no contrast agent used. To detect WMHs in the current investigation, we employed T1W and T2W dark fluid (FLAIR) images. All measurements were performed using a Philips Ingenia 3T scanner (Philips Healthcare, Best, The Netherlands) at the Semmelweis University Medical Imaging Centre, the Magnetic Resonance Research Centre (MRKK) in Budapest, Hungary and on a Siemens Magnetom Verio 3 Tesla workstation (Siemens Healthcare GmbH, Erlangen, Germany) at the Borsod County University Teaching Hospital, Miskolc. The following imaging parameters were used in the Philips scanner: TE/TR 140/9000 ms, flip angle 88°, 290 × 336 × 336 matrix, 0.8333 × 0.8333 in-plane resolution, 0.6 mm slice thickness. Twin pairs were always scanned on the same scanner either on the same day or, in the case of a few twin pairs, within a few weeks of each other. A single observer carried out all measurements in the study. This observer was not aware of zygosity or additional clinical information.

### 2.3. Image Processing

To convert the 3D T1-weighted pictures from DICOM (Digital Imaging and Communications in Medicine) format to NIfTI (Neuroimaging Informatics Technology Initiative; http://nifti.nimh.nih.gov/ (accessed on 30 August 2022)) format, we utilized a DCM2NII converter (http://www.mricro.com, mricron; Chris Rorden, Columbia, SC, USA, accessed on 19 September 2021). This picture format was used for all future image processing [21].

### 2.4. WMH Segmentation

**Figure 1:**
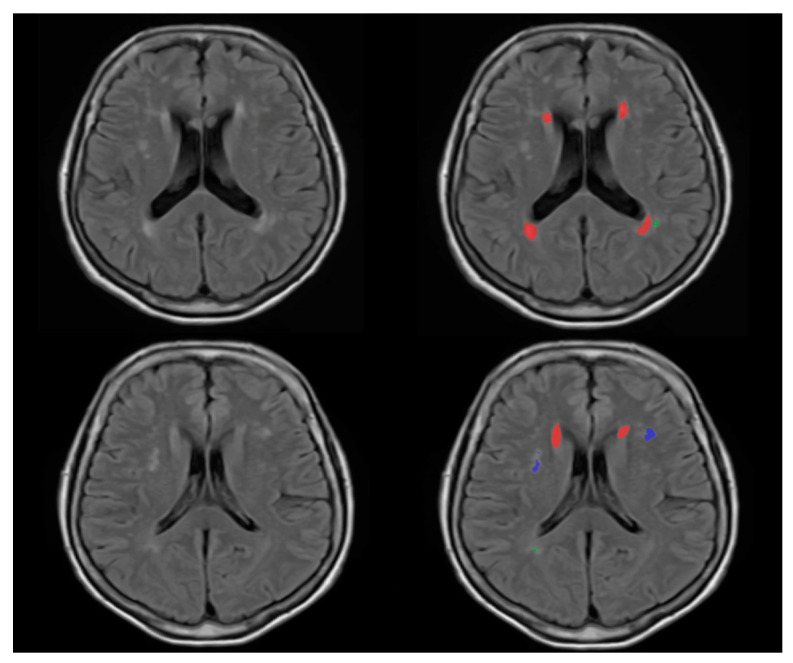
FLAIR MRI imaging of 65 years old female monozygotic twins (left column); the right side column pictures are the segmented version of the FLAIR images. The similarity of the anatomy and WMH of the cut is visible. According to our analysis, one twin has a total number of 17 WMHs (first row) and the other pair has a total number of 19 WMHs (second row). The red color represents the periventricular WMH and the blue and green colors represent the juxtacortical and deep white matter WMH—image from the Semmelweis University Medical Imaging Centre.

**Figure 2:**
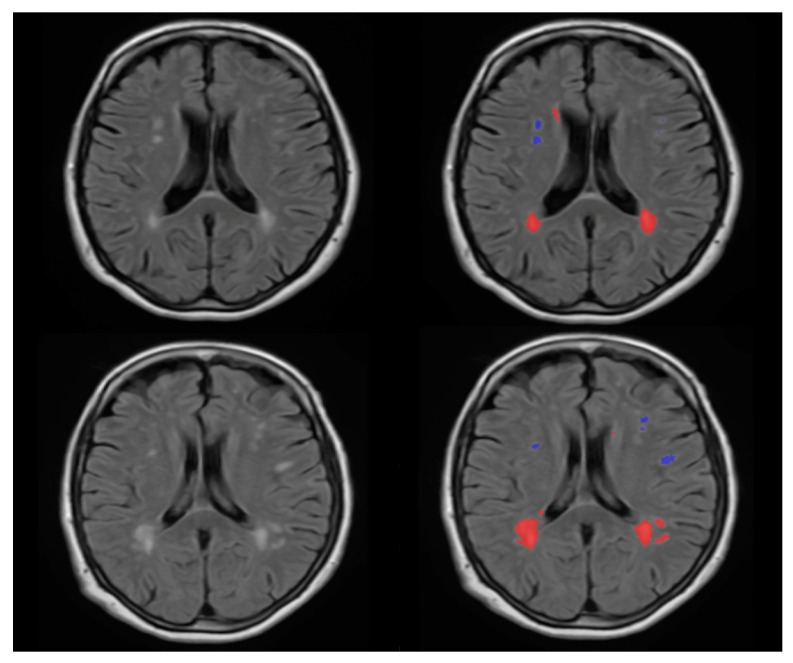
FLAIR MRI imaging of 69 years old female dizygotic twins (left column); the right side column pictures show the segmented version of the FLAIR images. The difference in the anatomy and number of WMH of the cut is visible in this image. According to our analysis, one twin has a total number of 24 WMHs (first row) and the other pair has a total number of 33 WMHs (second row). The red color represents the periventricular WMH and the blue color represents the juxtacortical WMH—image from the Semmelweis University Medical Imaging Centre.

We categorized the brain into four regions: periventricular, deep white matter, infratentorial and cerebellar; for the periventricular and deep white matter regions, we used the volBrain pipeline (https://www.volbrain.upv.es, accessed on 20 November 2020). VolBrain is MRI brain volumetry software that operates automatically and can offer brain structure volumes without human involvement; it has been developed by José V. Manjón (IBIME, UPV, Valencia, Spain) and Pierrick Coupé (LaBRI UMR 5800, Université de Bordeaux, CNRS, Paris, France). VolBrain employs a completely automated pipeline for volumetric brain analysis based on multi-atlas label fusion technology, which is capable of providing accurate volumetric information at various levels of detail in a short period of time [22]. WMH segmentation begins with image denoising, followed by inhomogeneity correction, spatial registration, intensity normalization and intracranial cavity extraction using the Montreal Neurologic Institute algorithm (MNI). The tissue is then segmented using a multi-template fusion atlas strategy based on a library created by manually segmenting 50 patients. All voxels that surpass a certain threshold are candidates for a lesion, The thresholding and voxel processing was carried out automatically by the volBrain program. Lastly, an automated report is generated, which contains the lesion load, the number of lesions in each class and screenshots of the processed images [23]. Figure 1 and Figure 2 demonstrate an example of MRI FLAIR sequence segmentation using volBrain.

### 2.5. Statistical Analysis

#### 2.5.1. Descriptive Statistics

The Shapiro-Wilk test was used to determine if continuous variables had a normal distribution. The means of the variables were compared between MZ and DZ twins using the independent samples *t*-test if they were determined to be regularly distributed. The BMI was one of these factors and was represented as a mean standard deviation (SD). The non-parametric Mann-Whitney U-test was used to compare variables that were not regularly distributed. This was applied to participant age, which was reported as the median, interquartile range (IQR). The Chi-square test was used to compare categorical variables such as sex, diabetes, smoking, hypertension, hyperlipidemia, chronic obstructive lung disease (COPD) and thyroid disorders reported as frequencies and percentages. A *p*-value of less than 0.05 was regarded as significant. The Statistical Package for the Social Sciences (SPSS) software was used for all descriptive statistical analyses (International Business Machines Corporation, IBM Corp. Released 2021. IBM SPSS Statistics for Macintosh, Version 28.0. Armonk, NY, USA: IBM Corp.).

#### 2.5.2. Heritability Analysis

ACE is a statistical model to assess and analyze a specific phenotype’s genetic and environmental contribution; using unobserved random variables, the influence of the genome and exposome may be explained. These variables include additive genetic (A), common (or shared) environmental (C) and unshared (or unique) environmental (E) components. The random variables are expected to be mutually independent within a single twin and to follow a conventional normal distribution within the same twin [24]. For the ACE analysis, version 2.19.5 of the OpenMx package for structural equations and other statistical modeling has been used under version 3.6.3 of the R programming language [25,26]. Common environmental characteristics allude to twins’ shared family environment, which includes similar childhood nutrition, parental smoking exposure, air pollution and even sharing a womb. Non-shared environmental variables, such as smoking behaviors, physical activity, occupational exposures and distinct diseases, are unique exposures and experiences for individual twins but not for their siblings [27].

The intra-pair correlations in MZ and DZ twins were compared to derive heritability estimates. If MZ twins had more significant intra-pair correlations than DZ twins, this suggested a genetic effect. If intra-pair correlations in both MZ and DZ twins were comparable, the variation was assigned to common environmental variables [28].

The variation was decomposed into additive genetic (A), common environmental (C) and unique environmental impacts, plus standard error, using univariate quantitative genetic modeling (E). All models were adjusted for age and gender. Because of confounding effects, dominant genetic variables (D) and common environmental factors (C) cannot be computed simultaneously using this approach. As a result, the best-fitting model (ACE) was used. For variables with minor genetic or common environmental impacts, a reduced AE or CE model was explored. To compare the models, a Chi-square test was used and *p*-values greater than 0.05 showed that there was no difference, supporting the usage of the more frugal AE or CE model.

## 3. Results

### 3.1. Descriptive Analysis

The median age was 46 and 64 years in the MZ and DZ groups, respectively. There was no significant difference between the two groups for BMI, smoking, diabetes, hypertension and hyperlipidemia. However, a significant difference was observed in age (*p* = 0.03) between the two groups. Table 1 shows the characteristics of the MZ and DZ twin study population. Due to the mean age difference in the MZ and DZ twins, we compared models where age was separately regressed out on MZ and DZ twins. This showed no significant difference compared to the base models, where regression on age and sex is carried out simultaneously in the MZ and DZ groups.

### 3.2. Results for WMH Count Measurement

For most variables, MZ twins had higher age- and sex-adjusted intra-pair correlation coefficients than DZ twins. Table 2 shows the results of the intra-pair correlation analysis of WMH count in different brain regions in MZ and DZ twins.

### 3.3. Univariate Model Analysis for the WMH Count in Different Brain Regions

Age- and sex-adjusted univariate analysis demonstrated heritability (A) for most variables. The analysis was run as a normal ACE model using normally distributed continuous variables for the total intracranial volume and total lesion count. Total intracranial volume was continuous and normally distributed; total lesion count was transformed as the log (total lesion count + 1), yielding a transformed variable, which the model analyzed. The rest of the variables, deep white matter, infratentorial, cerebellar and periventricular WMH count, were processed as count data.

We converted the numbers of deep white, infratentorial and cerebellar lesions to binary data. Patients with no WMH in that location remained at zero, whereas patients with counts greater than zero in that region were changed to one since there was insufficient data to discriminate the number of lesions in the model effectively.

Due to the higher number of WMH in the periventricular area compared to other locations, we analyzed the data as follows. In patients with zero, one, or two WMH in that location, the count was kept as is; in patients with more than three WMH, the data were binned as four. This whole procedure does not imply that the model believes there are four lesions but instead that it interprets the data as more than three. Generally, the AE model prevailed in the variables, indicating that the qualities are heritable to some extent.

The unique environmental variance was an essential contributor to all variables indicating the effect of environmental factors on the number of WMH. The findings of the univariate model heritability study for the count of the WMH in various brain areas using volBrain in twins are shown in Table 3.

## 4. Discussion

This study aimed to investigate the genetic contribution to WMH count in the four regions of periventricular, deep white matter, infratentorial and cerebellar of the brain, using 3T MRI in middle age and healthy groups of twins. According to our sample, WMH counts are heritable to some degree and the heritability of total lesion count reinforces the heritability of the separate regions. The A value is high in the areas being supplied by the posterior brain circulation (infratentorial region) and the E value is low compared to the areas provided by the anterior brain circulation (supratentorial region), meaning the effect of genetics on the posterior brain circulation and its territory is higher than the anterior brain circulation and its territory. The impact of the environmental factors is more significant in the anterior brain circulation than in posterior brain circulation and its territory.

Previous studies proved that WMH is a highly heritable [29,30,31,32] in a sample of female and male older adult twins, suggesting a substantial genetic component. Females have greater heredity than males in all cerebral lobes, notably the periventricular area, which has a low heritability in men. In both sexes, the heritability of deep WMH reduced with age, particularly beyond the age of 75 [29]. The San Antonio Family Study discovered that the shared genetic variability across subcortical (DWMH) and ependymal (PWMH) volume volumes were 21%, suggesting strong pleiotropy [33]. The Cohorts for Heart and Aging Research in Genomic Epidemiology (CHARGE) collaboration revealed six new risk-associated genes, including a novel locus on chromosome 17, that collectively accounted for 4% to 8% of the WMH burden [34]. Carmelli et al. reported a heritability of 73% for WMH volumes, which was lowered to 71% when age and head size were considered. The volumes of WMH were substantially associated with MZ pairings and the correlations were more significant in MZ couples than in DZ pairs [35]. In addition to previously hypothesized ischemia processes, a multi-ethnic investigation involving European, Asian, African and Hispanic patients discovered four additional genetic loci implicating inflammatory and glial proliferative pathways in the development of WMH [36].

Given that our cohort included middle-aged twins, the effect of environmental factors on the prevalence of WMH would be more visible, potentially outweighing the impact of genetics on the prevalence of WMH. Furthermore, using a 3T MRI scanner instead of a classic 1.5 T detected the WMH with greater precision, which may reveal the full effect of environmental factors. Due to the aforementioned factors, our findings revealed a moderate genetic influence on the occurrence of WMH, as contrasted to other studies in this field, which revealed a more substantial genetic influence on the occurrence of WMH [29,30,31,32,33,34,35,36].

The general heritability of WMHs shown in our research and other investigations suggests that the individual manifestation of WMH has a substantial genetic component. This study raises intriguing issues about the possible origins of WMHs. As previously stated, both age and the presence of cerebrovascular illness affect WMH. Therefore, high heritability estimates for WMHs may imply pleiotropy with complicated aging characteristics, complex cerebrovascular risk factors, or both. Notably, linkage studies may reveal chromosomal areas or candidate genes implicated in the genetic control of WMHs [8]. The genetic investigation of white matter lesions yielded few results, with few candidate genes examined and only one genome-wide association study conducted. A few linkage investigations have been performed, providing suggestive evidence of genetic linkage for white matter lesions, but the actual genes involved have yet to be discovered. The angiotensinogen gene, situated on chromosome 1q42 and with a significant number of polymorphisms, had the most consistent findings [37].

According to our study, environmental factors moderate the number of WMHs, especially in the supratentorial regions. In general, aging and major cardiovascular risk factors such as diabetes mellitus and smoking increase the WMH in patients [9,38,39,40]. This can influence the development of carotid plaques, which might influence the development of WMH in the supratentorial region in comparison with the vertebral arteries, which are less frequently affected by atherosclerosis. Blood glucose level and prediabetic state are risk factors for WMH development. According to one study, a high 2-h blood glucose concentration in the OGTT, but not fasting blood glucose (FBG) levels, may be an independent risk factor for developing WMHs, providing insight into the need for enhanced preventative interventions in patients at risk of WMH-associated morbidity [13]. According to another study, impaired FBG levels are also linked to increased WMH burden in older community-dwelling individuals with or without T2D [41]. Some studies revealed a strong correlation between DM and cortical atrophy but not WMH [42]. On the contrary, other studies showed significant associations between type 2 DM and the prevalence of WMHs [12,43]. Obesity and BMI were also found to be influential on WMH development, with a selective increase in the WMH load in the deep white matter of obese patients with significant visceral fat deposition, irrespective of typical obesity-related comorbidities such as hypertension. According to mediation studies, visceral obesity may contribute to deep white matter lesions by increasing proinflammatory cytokines, implying a patho-mechanistic relationship [15,44,45]. Among one of the most common habits of society, smoking is one of the factors related to the progression of WMH; according to one study in 23% of smokers, WMH progression was detected; increasing pack-years of smoking increases the risk of WMH progression; no association was seen between time since quitting and age at smoking initiation. However, according to one study, the effect of cigarette smoking on the brain varies with age [14,46].

Our findings indicate that, in addition to the moderate hereditary effect on the incidence of WMH, environmental factors play an important role in the occurrence of WMH, particularly in the territory of anterior cerebral circulation. Aside from the research on epigenetic modification that should be carried out to reduce the genetic effect, environmental improvements such as diabetes management and prevention, weight loss and physical activity, smoking cessation, hypertension management and prevention and stress management will have a significant impact on the number and impact of WMH in the population.

Our study’s limitations must be addressed. There is a possibility of procedure and measurement bias since the MRI pictures were acquired using two separate MRI machines in two distinct locations. Additionally, since volBrain is entirely automated, our segmentation could not be manually adjusted, which has the benefit of comparable data without observer bias but also has the downside of being unable to separate WMHs from possible artifacts. We discovered a statistically significant difference in the average age of the MZ and DZ groups which might bias the results. Our findings may potentially be skewed by our group’s very young average age, since age corresponds with the frequency and volume of WMH lesions, resulting in fewer lesions in our sample. Comorbidities may also have a role in the low total number of lesions. The lack of a proper automated program for segmentation and counting infratentorial WMH was an additional limitation.

## 5. Conclusions

We discovered that WMH counts are heritable to a moderate extent in our middle age healthy sample and that the heritability of the overall lesion count strengthens the heritability of the distinct areas. The degree of this heritability is different in different brain regions; overall, the genetic component influencing the count of WMH in the infratentorial region is stronger than the effect of genetics on the count of WMH in the supratentorial areas. On the other hand, environmental factors are more prominent in the areas supplied by the anterior brain circulation than those supplied by the posterior brain circulation. Our findings suggest that these features also have a hereditary component in middle aged individuals; nevertheless, we were unable to assess the heritability of many WMH traits due to the low prevalence of lesions in this relatively young and healthy group. Additional research with more significant populations is required to corroborate our findings.

## Figures and Tables

**Table 1:** Characteristics of the MZ and DZ twin study populations, Body mass index (BMI) and other continuous variables with a normal distribution are represented by the mean and standard deviation and those with a non-normal distribution are represented by the median, interquartile range (age). † The results of the non-parametric Mann-Whitney U-test are displayed. For all other continuous variables, the independent-sample *t*-test was used to determine the *p*-value. *p*-values are obtained for dichotomous variables using the Chi-square test. Asterisks (*) are used to indicate significant results.

Characteristics	Total (*n* = 120)	MZ (*n* = 86)	DZ (*n* = 34)	*p*-Value
Zygosity (*n*MZ:*n*DZ)	86:34	-	-	-
Sex (male:female)	33:87	22:64	11:23	0.45
Age (years)	50 ± 26.5	46 ± 23	64 ± 29	0.03 ^†^*
BMI, kg/m^2^	24.4 ± 4.3	24.3 ± 4.6	24.7 ± 3.4	0.68
Smoking *n*(%)	12(14.0)	9(15.3)	3(11.1)	0.75
Diabetes *n*(%)	7(8.1)	4(6.8)	3(11.1)	0.67
Hypertension *n*(%)	24(27.9)	15(25.4)	9(33.3)	0.45
Hyperlipidemia *n*(%)	22(25.6)	16(27.1)	6(22.2)	0.79
COPD *n*(%)	9(7.5)	7(8.14)	2(5.9)	0.67
Thyroid disorders *n*(%)	24(20)	16(18.6)	8(23.5)	0.54

**Table 2:** Intra-pair correlation coefficients of WMH count in the different brain regions in MZ and DZ twins. Results and 95% confidence intervals were reported (95% CI). rMZ: intra-pair correlation coefficient in monozygotic twins, rDZ: intra-pair correlation coefficient in dizygotic twins.

Variable	rMZ	rDZ
Total WMH count	0.466 (0.195 0.671)	−0.025 (−0.451 0.421)
Deep white matter WMH count	0.482 (0.038 1)	0.093 (−0.631 1)
Infratentorial WMH count	0.739 (0.371 1)	0.390 (−0.32 0.686)
Cerebellar WMH count	0.537 (0.041 0.84)	0.372 (−0.348 0.522)
Periventricular WMH count	0.473 (−1 1)	0.190 (−1 1)

**Table 3:** Age- and sex-adjusted univariate analysis of the count of WMH in the different brain regions using volBrain in twins. The results were presented together with 95% confidence intervals (95% CI). A stand for heredity, C for shared environmental variation and E for unique environmental variance.

Variable	A	C	E	*p*-Value
Total lesion count	0.403 (0.156, 0.6)	0	0.597 (0.4, 0.844)	1
Deep white matter lesion count	0.450 (0, 0.766)	0	0.550 (0.234, 1)	1
Infratentorial lesion count	0.727 (0.371, 0.919)	0	0.273 (0.081, 0.629)	1
Cerebellar lesion count	0.555 (0.201, 1)	0	0.445 (0, 0.799)	0.776
Periventricular lesion count	0.472 (0.145, 0.712)	0	0.528 (0.288, 0.855)	1

## Data Availability

Data are available upon request.
